# Detection of cadmium in soils using laser-induced breakdown spectroscopy combined with spatial confinement and resin enrichment

**DOI:** 10.1039/c8ra07799a

**Published:** 2018-11-27

**Authors:** Xinglan Fu, Guanglin Li, Hongwu Tian, Daming Dong

**Affiliations:** College of Engineering & Technology, Southwest University 216 Tiansheng Road, Beibei Chongqing 400716 PR China; Beijing Key Laboratory of Digital Plant, National Engineering Research Center for Information Technology in Agriculture, Beijing Academy of Agriculture and Forestry Sciences Beijing 100097 China damingdong@hotmail.com dongdm@nercita.org.cn

## Abstract

The determination of heavy metals in soils is of great significance for the monitoring and control of environmental pollution. However, it is hard to realize fast and *in situ* measurements. Laser-induced breakdown spectroscopy (LIBS) is an effective method for element detection in soils, but its detection limit cannot meet the requirements of the control of soil pollution. In addition, it usually suffers splash problems and needs complex pretreatment processes before measurement. In this study, we developed a new method for the determination of cadmium in soils using LIBS. We improved the sensitivity of common LIBS, while avoiding splash problems and without complex pretreatment processes. The LIBS signal is enhanced in two ways. Firstly, the heavy metals were enriched by the cation exchange resins. And then, the LIBS signal levels were further enhanced by a sample container with spatial confinement. During this process, the soil only needs to be treated with water to achieve slurry status, rather than any complex pretreatments. We demonstrated that the detection limit for cadmium in soils is 0.132 mg kg^−1^ using this method.

## Abstract 2

The determination of heavy metals in soils is of great significance for the monitoring and control of environmental pollution.

## Graphical abstract

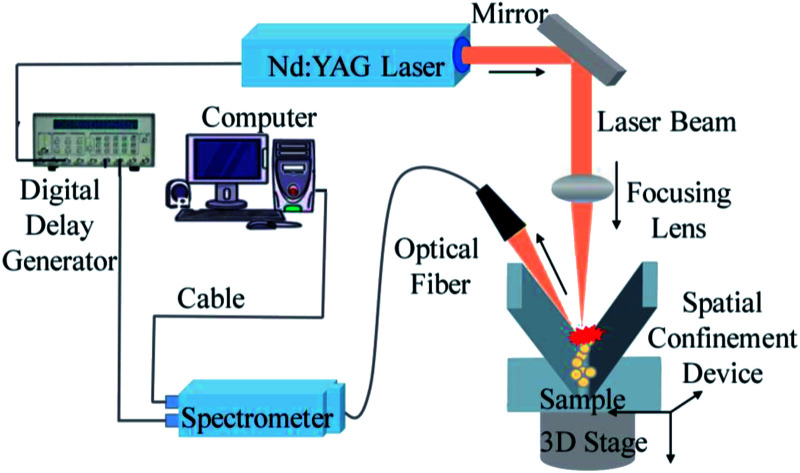

## 1. Introduction

Soil is an important part of the ecological environment, and is a major resource contributing to human survival and development.^1^ Along with urbanization, industrialization, mineral resource development and use of metal smelting, the application of sewage irrigation and fertilizers as well as other human activities have contributed a variety of heavy metal pollutants through various channels to the soil. This has caused increasingly serious heavy metal pollution of soils.^2^ Any introduction of this heavy metal pollution into the human body through the food chain will cause harm to human health.^3–6^ Therefore, an effective detection of heavy metal content in soils and the control of excessive heavy metal source emissions are both important means to improve the safety of agricultural products and food.

Conventional analytical techniques for soils involve inductively coupled plasma atomic emission spectrometry (ICP-OES), inductively coupled plasma mass spectrometry (ICP-MS), laser ablation-inductively coupled plasma-mass spectrometry (LA-ICP-MS), high performance liquid chromatography (HPLC), atomic fluorescence spectrometry (AFS), atomic absorption spectroscopy (AAS) and X-ray fluorescence analysis.^7–9^ These methods are widely used in soil for microelement analysis because of their high sensitivity and repeatability.^10^ However, these methods require time-consuming sample preparation and cannot be applied *in situ* for rapid determination of pollution levels.^11,12^

Researchers have proved that laser induced breakdown spectroscopy (LIBS) is a competitive spectroscopy technology based on plasma spectral analysis from samples. When the laser ablated and excited the sample surface, the plasma created from the sample contains elemental information about the sample by the optical emission.^13,14^ LIBS can measure various substances, including solid, liquid, colloid, and gas phases, and provides a nondestructive, rapid and *in situ*, simultaneous multi-elemental analysis. As a result, LIBS has been applied successfully to rapid on-site analysis of soil, food, art, and explosives.^15,16^ LIBS has the advantages of simple sample preparation, fast monitoring, simultaneous detection of multiple elements, and high sensitivity, making it ideal for *in situ* monitoring of multiple heavy metals in soils.^17,18^ Researchers have already conducted many studies on the detection of heavy metals in soils using conventional LIBS.^19–21^ For example, LIBS combined with RBF neural network used to analyze Cd in soils, and obtained the LOD of Cd was 16.5 mg kg^−1^.^22^ The spectral characteristics of Cd were analyzed by LIBS in pellet soil samples, yielding the calculated detection limits were 1.3, 3.6 and 4.0 μg g^−1^ for Cd II 214.441 nm, Cd II 226.502 nm and Cd I 228.802 nm, respectively.^23^ More recently, researchers have used LIBS combined with new technologies to improve soil heavy metal detection capabilities. In particular, the detection sensitivity of Cu and Ag in soils was improved by using microwave-assisted LIBS technology, whereby the detection limits of these two elements were lowered to 30 mg kg^−1^ and 23.3 mg kg^−1^, respectively.^24^ LIBS has also been combined with laser-induced fluorescence technology could detect the limits of Cd elements in soils was 0.3 μg g^−1^.^25^ In addition, soil heavy metal Pb were detected using dual-pulse LIBS and spatial confinement, reducing the detection limits of Pb respectively to 20 mg kg^−1^, and 10 mg kg^−1^.^26,27^ However, the control standard of Cd in soils is below 0.2 mg kg^−1^ in China, while the sensitivity of above methods cannot reach this concentration. Furthermore, there are several problems with these methods: pre-treatment processes, such as grinding and drying, are still required; soils are prone to splatter during measurement; and these low detection limits are difficult to achieve in practice.

The ion-exchange resin can enrich heavy metals and commonly used to remove heavy metals from wastewaters.^28–30^ In this paper, we tried to improve the sensitivity convenience of common LIBS system by combing ion-exchange resin with spatial confinement. We will demonstrate the following advantages of the method: (1) the sensitivity of LIBS will be enhanced by both spatial confinement and the enrichment of ion-exchange resin, which meets the requirement for the detection of cadmium in soils; (2) the complex pretreatment processes will be simplified through transfer cadmium from the soils into the ion-exchange resin; (3) the splash problems that troubles soil measurement using LIBS will be avoided.

## 2. Materials and methods

### 2.1 Materials

We collected the soil samples from field. Then we send them to Beijing Research Center for Agriculture Standards and Testing for testing. The results showed that there were no Cd in soils. The (Cd(NO_3_)_2_) solution with concentration of 100 μg ml^−1^ were prepared using the analytically grade (>99.99%) solid powder of (Cd(NO_3_)_2_). The cation exchange resin ECS60 was obtained from Hangzhou Yongzhou Water Treatment Technology Co., Ltd. The properties of the resins are shown in Table 1. The spatial confining device was a V-shaped stainless-steel plate having a 60° angle.

**Table 1:** Characteristics of ECS60 cation exchange resins

Characteristics	Values
Active group	–CH_2_N–(CH_2_COONa)_2_
Matrix	Macroporous styrene-divinylbenzene
Ionic forms as shipped	Na^+^
Physical form	Spherical beads
Mean particle size (mm)	0.315–1.25
pH range	6–11
Total exchange capacity	≥1 meq. L^−1^

### 2.2 Instruments

**Fig. 1:**
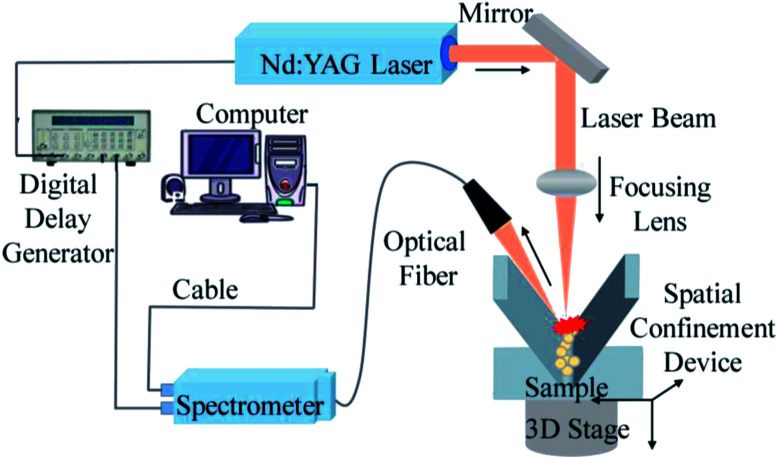
The schematic diagram of the LIBS system.

The LIBS system comprised a laser, a spectrometer, a motor-controlled high-precision three-dimensional platform, and a digital signal delay generator. Fig. 1 shows the structure of the experimental system. The laser was a Q-switched Nd:YAG Laser System (Beamtech Optronics Ltd., Beijing, China) with wavelength 1064 nm. The pulse width was 3–5 ns with the repetition frequency of 20 Hz. The maximum energy output of the pulse was 200 mJ and the exit angle was within 1 mrad. The laser was collimated by focusing lens and focused on sample surface. The laser-induced plasma light signal was collected using an optical fiber and transmitted to the spectrometer (HR2000+; Ocean Optics Co., Largo, FL, USA). The spectral range of the spectrometer was from 200 nm to 1100 nm. A high-precision three-dimensional platform controlled by a stepper motor performed precise 3D adjustments, measuring different positions of each sample. A digital signal delay generator was used to generate a trigger delay between spectrometer acquisition and the laser. The signal delay device, three-dimensional platform, and spectrometer were all controlled by the same computer.

In this experiment, the laser pulse energy was set to 160 mJ, the spectral range of spectrometer was set to from 200 nm to 1000 nm, and the integral time was set to 2 ms. This study focused on the atomic emission peaks of Cd. Before the experiment, a background spectrum was collected by the spectrometer, which was deducted from all subsequent measurements.

### 2.3 Method

The experimental method was divided into the following steps:

#### (1) Contaminated soil samples preparation

The soil samples were mixed with different volume of (Cd(NO_3_)_2_) solution. We then stirred it evenly to prepare Cd contaminated soil samples. Finally, Cd contaminated soil samples were dried at room temperature.

#### (2) Traditional pellet soil preparation

To prepare a traditional tablet, the contaminated soil samples were compacted into a cylindrical pellet with 13 mm diameter using a mechanical press (with *P* = 10 t for 7 s). The concentration of Cd in this soil ingot was 100 mg kg^−1^.

#### (3) Resin-enriched sample preparation

**Fig. 2:**
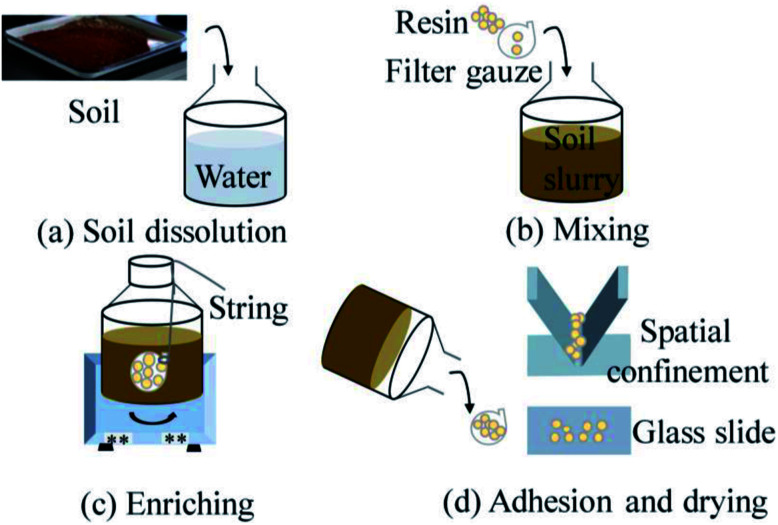
The procedure of resin enrichment contains four steps (a) soil dissolution, (b) mixing, (c) enriching, (d) adhesion and drying.

The resin enrichment sample preparation contains four steps, as shown in Fig. 2. First, the contaminated soil samples were remixed with deionized water, while water: soil ratio was 1 : 1. The concentration of the Cd elements in slurry were 0.667–13.33 mg kg^−1^. Then we put resins into a filter gauze and mixed it with slurry by shaking in an oscillator. After the mixture for 150 min at a speed of 270 rpm,^31^ we took the filter gauze out from the slurry. Next, we adhered resins on a glass substrate and a spatial confinement device, respectively. Finally, the samples were dried, measurements were carried out using the LIBS.

## 3. Results and discussion

### 3.1 Resin enrichment and spatial confinement to enhance sensitivity

**Fig. 3:**
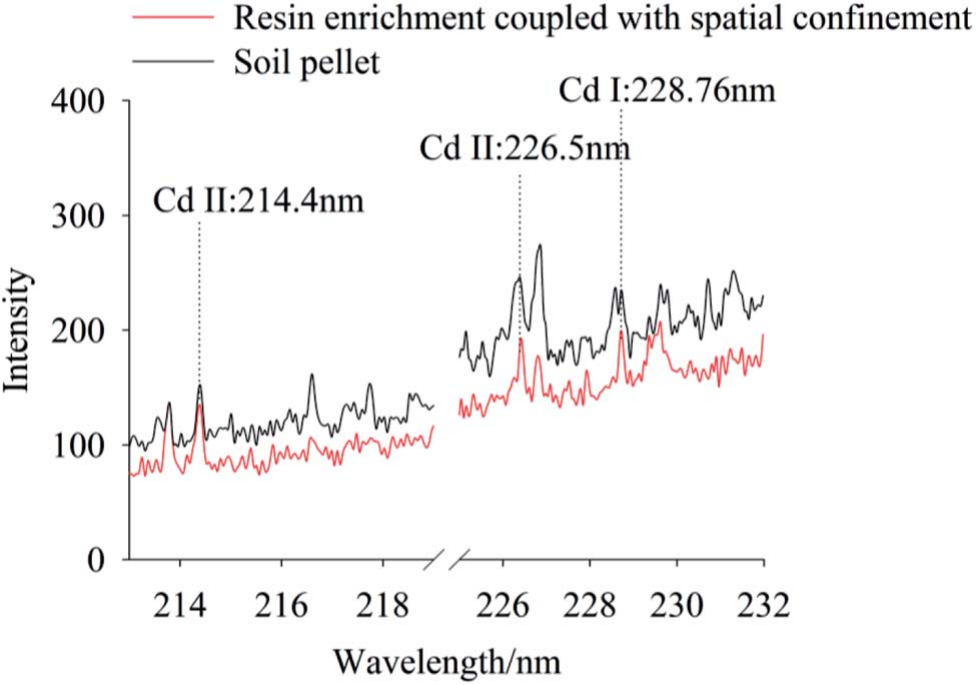
The LIBS spectra of Cd polluted soil using the traditional pellet soil method and our method, while the Cd concentration is 100 mg kg^−1^ for the former and 0.667 mg kg^−1^ for the latter. (To clearly show the differences among these spectra, we parallel moved each spectrum. The same operation were also taken in Fig. 4–8.)

To compare signal characteristics, samples prepared using the traditional tablet, resin enrichment coupled with spatial confinement method were measured using the LIBS system, with Cd concentration of 100 mg kg^−1^ and 0.667 mg kg^−1^, respectively. In this experiment, the characteristic spectral peaks of Cd II occurred at 214.4 nm, 226.5 nm and Cd I was at 228.76 nm, as shown in Fig. 3. It can be seen the spectral lines of Cd at 214.4 nm, 226.5 nm and 228.76 nm can be detected both by traditional tablet method (100 mg kg^−1^) and by resin enrichment coupled with spatial confinement (0.667 mg kg^−1^). However, Cd concentration in the pellet soil sample were 150 times higher than in the resin enrichment coupled with spatial confinement sample. Clearly, the resin enrichment coupled with spatial confinement method used in this study improved the detection capability of the LIBS technology obviously. This is because the resin matrix of styrene and the cross-linker divinylbenzene produce a polymerization reaction to form a long backbone structure with crosslinks. Given the matrix had a simple spectrum and Cd was enriched in the resin, the extraction and analysis of the spectral peaks of Cd was facilitated. Under confinement, the Cd signal strength also was enhanced. Compared with the traditional tablet method, these newer methods greatly simplified the pretreatment of the soil and avoiding contamination of the optical lens by soil splattered during analysis. In particular, the space confining device was simple to operate and required no structural modification of the instrument.

### 3.2 The effect of spatial confinement in measurement

**Fig. 4:**
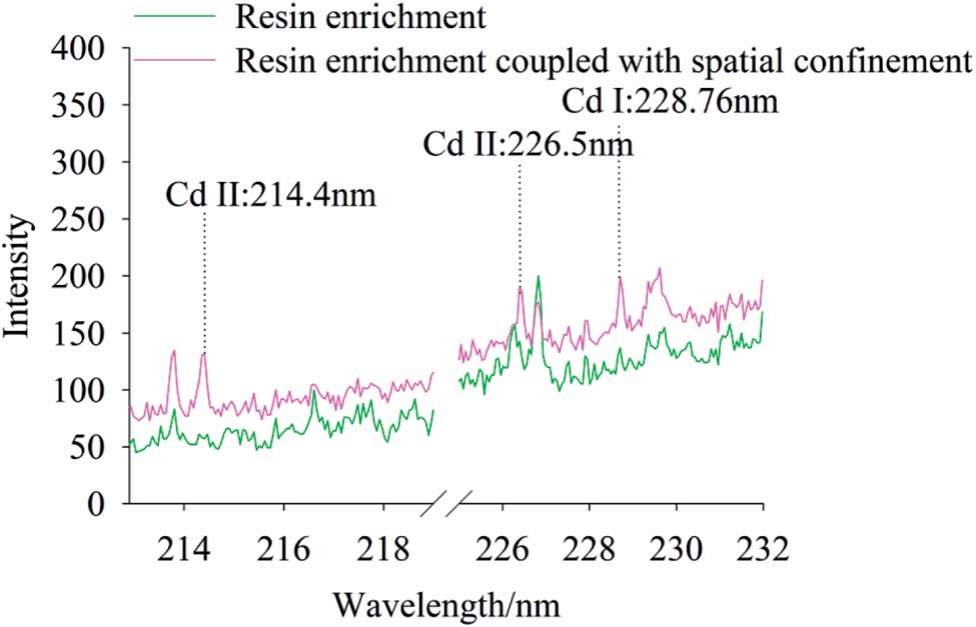
The LIBS spectra of Cd polluted soil using resin enrichment method coupled with and without spatial confinement, while Cd concentration was 0.667 mg kg^−1^.

**Fig. 5:**
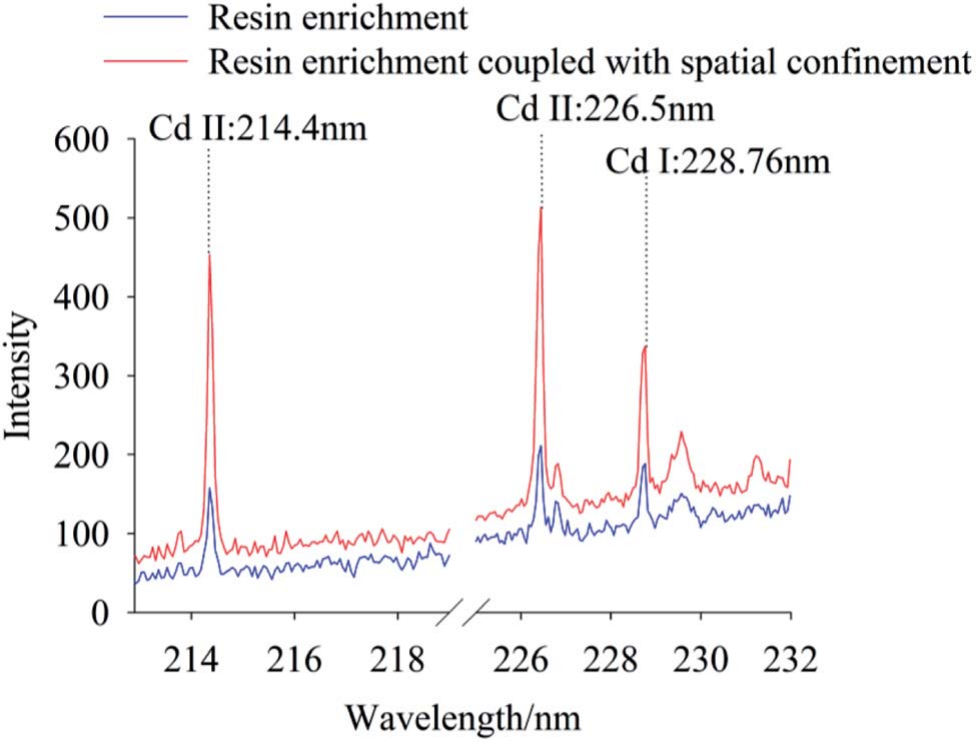
The LIBS spectra of Cd polluted soil using resin enrichment method coupled with and without spatial confinement, while Cd concentration was 6.67 mg kg^−1^.

The effect of resin enrichment coupled or not with spatial confinement on the characteristic peaks of Cd in soils were compared for Cd concentrations of 0.667 mg kg^−1^ and 6.67 mg kg^−1^. Fig. 4 and 5 show the spectra of resin enrichment with and without spatial confinement. It should be noted that resin enrichment coupled with spatial confinement detected Cd II at 214.4 nm, 226.5 nm and Cd I at 228.76 nm, showing obvious characteristic spectral peaks at both concentrations. When the Cd content was 0.667 mg kg^−1^, the resin enrichment without spatial confinement only detected a slight peak at 228.76 nm, while peaks at 214.4 nm and 226.5 nm were not detected. When the Cd content was 6.67 mg kg^−1^, the emission lines of Cd were all significantly enhanced. This is because of the effects of spatial confinement of the laser plasma, which under the excited states enhanced binding effects, reflecting back the shock wave and compressing the plasmas plume, resulting in increased signal strength.^32^

### 3.3 Quantitative measurement of heavy metals in soils

**Fig. 6:**
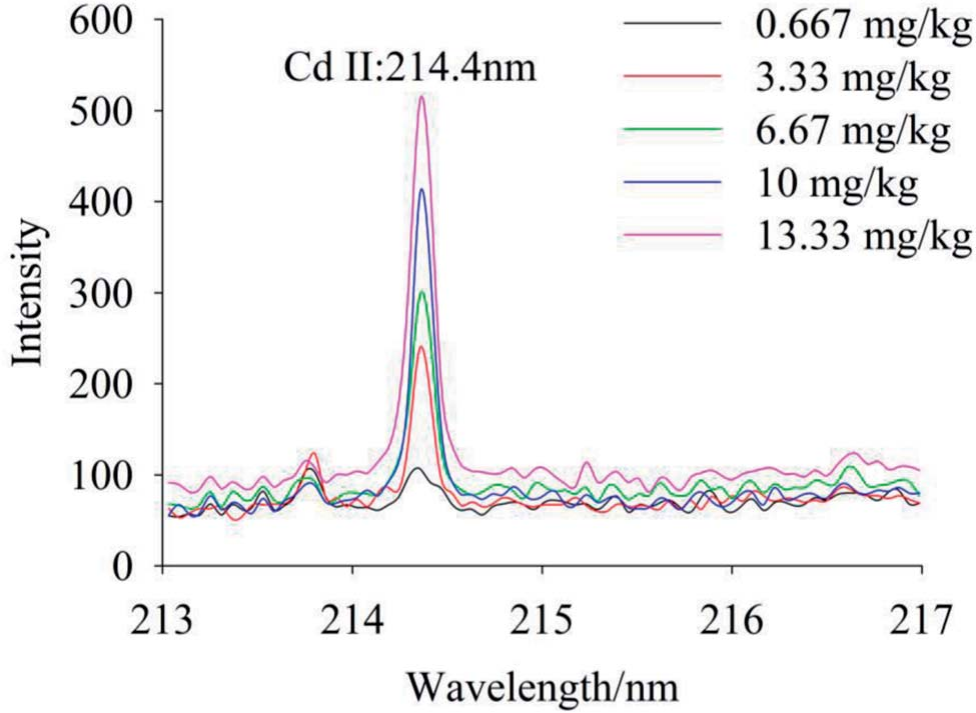
The spectral peaks of Cd at 214.4 nm, while Cd concentration was 0.667–13.33 mg kg^−1^.

**Fig. 7:**
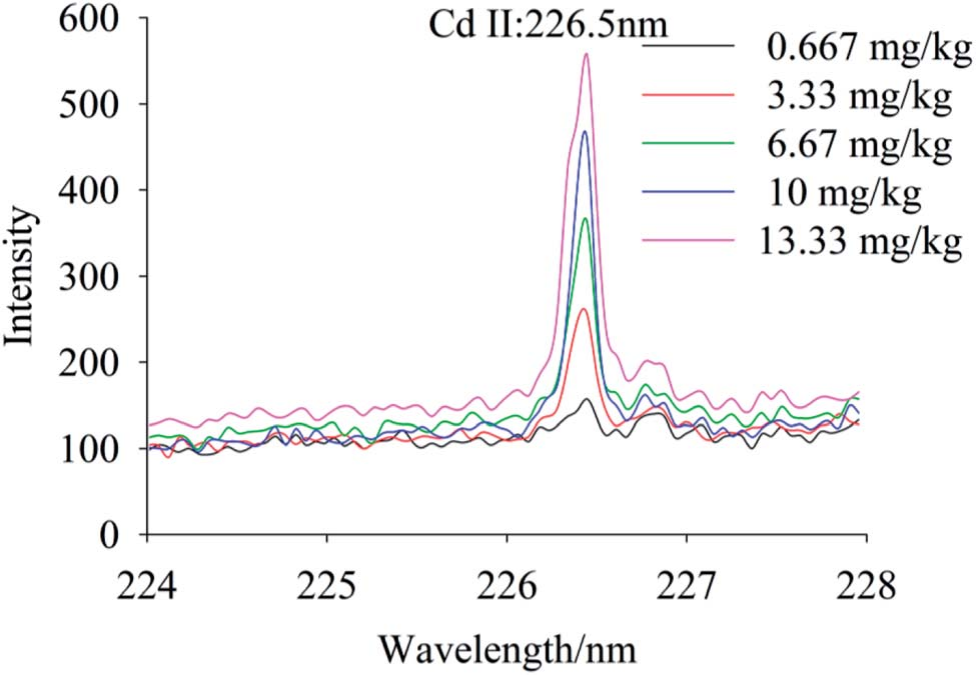
The spectral peaks of Cd at 226.5 nm, while Cd concentration was 0.667–13.33 mg kg^−1^.

**Fig. 8:**
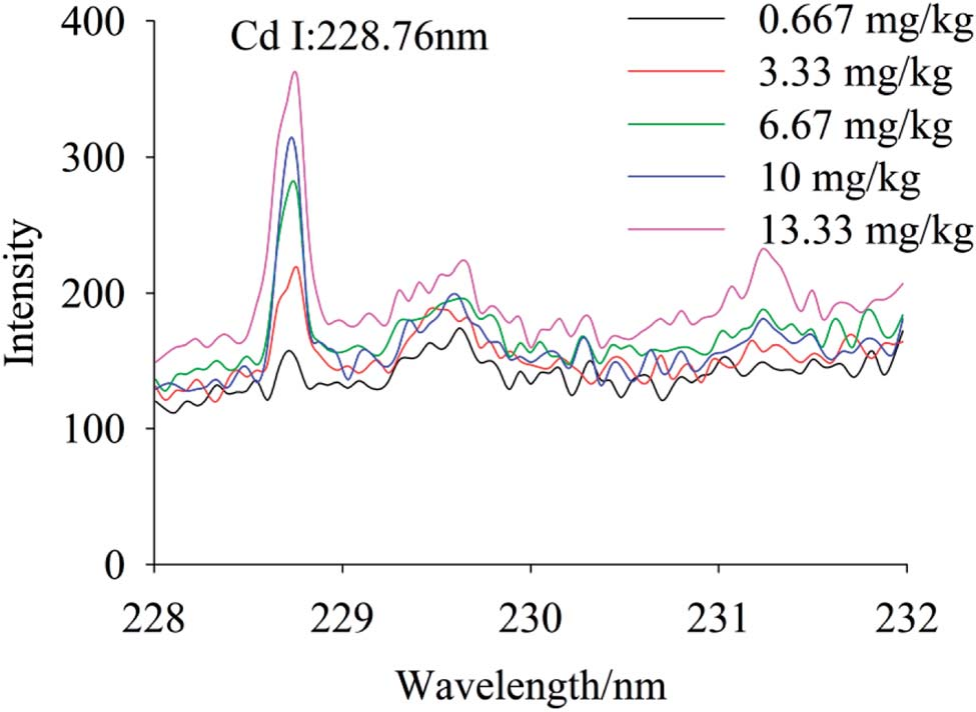
The spectral peaks of Cd at 228.76 nm, while Cd concentration was 0.667–13.33 mg kg^−1^.

**Fig. 9:**
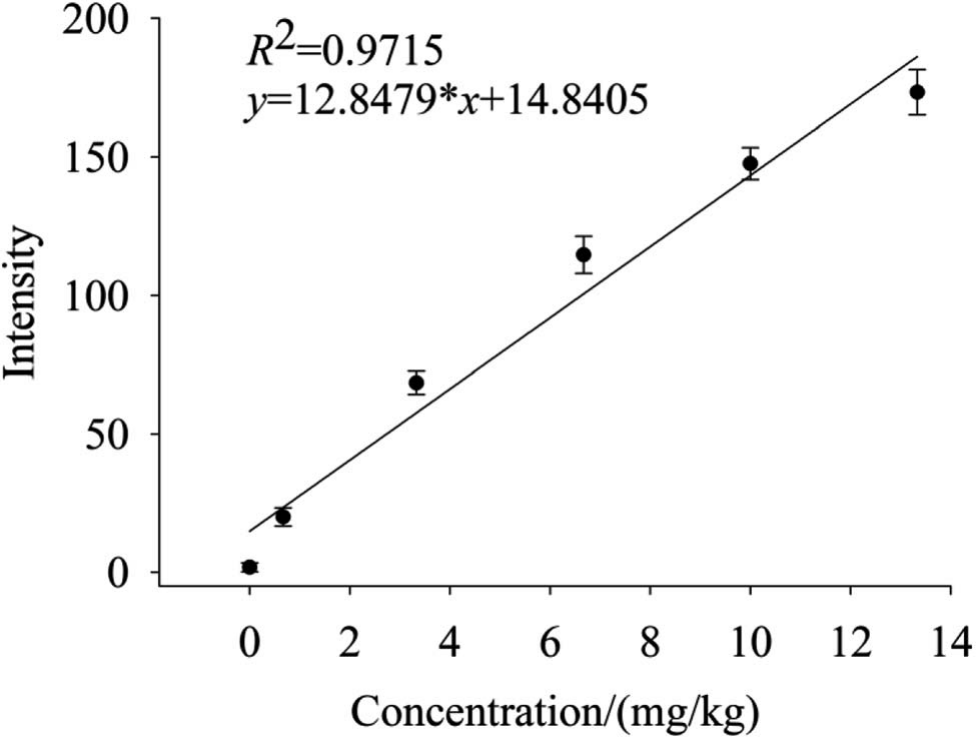
Calibration curve of Cd at 226.5 nm, while Cd concentration of 0–13.33 mg kg^−1^.

To quantify different Cd contents in soils, samples were prepared for LIBS measurement using spatial confinement combined with resin enrichment with Cd contents ranging from 0.667 mg kg^−1^ to 13.33 mg kg^−1^. The characteristic spectral peaks of Cd at 214.4 nm, 226.5 nm, 228.76 nm are shown in Fig. 6–8, respectively. Obviously, as the concentration of Cd in soil samples increased, the intensity of the characteristic peaks of Cd also increased, reflecting the positive correlation between Cd concentration and signal intensity. Based on these results, we selected the Cd peak at 226.5 nm for quantitative analysis. A calibration curve was determined (Fig. 9), which indicates that Cd concentration in the soil had a linear relationship with characteristic peak intensity, with a coefficient of determination of 0.9715.

### 3.4 Limit of detection

The limit of detection was 0.132 mg kg^−1^, which is determined by the ratio of the standard deviation of triplicate blank samples to the slope of the calibration curve.^33^ With the aid of high efficiency of absorption by resin and spatial confinement for plasmas plume, our method has largely improved the LOD for Cd, comparing with the conventional methods (which is 16.5 mg kg^−1^,^22^ 3.6 μg g^−1^ (ref. 23) and 0.3 μg g^−1^,^25^ respectively). The experiment results proved that the resin enrichment combined with spatial confinement method is much effective than the traditional table method. Our method satisfied the environmental quality standards of soil pollution (0.2 mg kg^−1^).

## 4. Conclusion

This experimental study demonstrated that LIBS measurements carried out on Cd in soil samples provided reliable quantitative results. Effects of the traditional tablet method, resin enrichment, and space confinement coupled with resin enrichment on the spectral characteristics of Cd peaks were analyzed. We conclude that: (1) compared with the resin enrichment method, spatial confinement coupled with the resin enrichment method markedly enhanced the signal of Cd from soil samples; (2) the resin enrichment method simplified pretreatment of samples and solved the problem of soil splash; and (3) the Cd II at 226.5 nm was selected for quantitative analysis, yielding a coefficient of determination of 0.9715 and a limit of detection of 0.132 mg kg^−1^. Heavy metals in soils can be divided into several forms-water-soluble metals, exchangeable metals, metals precipitated as inorganic compounds, metals complexed with large molecular-weight humic materials, metals adsorbed or occluded to precipitated hydrous oxides, metals precipitated as insoluble sulfide and metals bound within the crystalline lattice structure of primary minerals. Among them, water-soluble cadmium was the most mobile and plant available. The activities of exchangeable cadmium were much higher and its binding capacity with solid phase absorption medium was much weaker than others. The exchangeable cadmium was converted relatively easily to water-soluble cadmium. The water-soluble cadmium and exchangeable cadmium were the important source of cadmium contamination in soil, flora and fauna and could reflect soil pollution level in a way.^34^ Consequently, we used the resin to enrich these two forms of cadmium in soils and used LIBS to detect cadmium in our experiment. The results of this work provide a more accuracy method to detect trace heavy metals in soils using LIBS.

## Conflicts of interest

There are no conflicts to declare.

## Supplementary Material
